# Correlating the Gut Microbiota and Circulating Hormones with Acne Lesion Counts and Skin Biophysical Features

**DOI:** 10.3390/microorganisms11082049

**Published:** 2023-08-09

**Authors:** Raja K. Sivamani, Jessica Maloh, Yvonne Nong

**Affiliations:** 1 Integrative Skin Science and Research, Sacramento, CA 95815, USA; 2 Department of Dermatology, University of California-Davis, Sacramento, CA 95616, USA; 3 College of Medicine, California Northstate University, Elk Grove, CA 95757, USA; 4 Pacific Skin Institute, Sacramento, CA 95815, USA

**Keywords:** acne vulgaris, gut, microbiome, hormones, skin, biophysical features

## Abstract

Acne vulgaris is a common inflammatory condition that is multi-factorial and impacted by both intrinsic and extrinsic features. Several previous studies have assessed for correlations between factors such as circulating hormones, stress, or the microbiome. However, there have not been any correlations specifically against lesion counts or differentiating correlations between inflammatory and non-inflammatory lesion counts. Here, we correlate several factors against acne lesions. Twenty men and women with mild to moderate acne were recruited, and their hormonal levels and their gut microbiome were collected and correlated against their inflammatory and non-inflammatory lesions of acne. Facial non-inflammatory lesions were weakly correlated to sebum excretion rate and weakly inversely correlated to forehead and cheek hydration. We examined stress through the use of a normalized peak-to-trough ratio (higher numbers indicated less stress), which correlated with skin hydration and inversely correlated with sebum excretion rate. Sebum excretion rate was weakly correlated to testosterone levels, and facial hydration correlated with estradiol levels. Correlations with the gut microbiome showed differential correlations with inflammatory and non-inflammatory lesions, with *Clostridium sp AF 23-8* correlating to inflammatory lesion counts, while *Actinomyces naeslundii str Howell 279* correlated to non-inflammatory lesions. Overall, measures of stress and circulating hormones correlate to skin biophysical properties and acne lesion counts. Also, different gut bacteria correlate with either inflammatory or non-inflammatory lesion counts. We hope that our findings stimulate further work on the gut–mind–stress–skin axis within acne.

## 1. Introduction

Acne vulgaris affects over 85% of adolescents and young adults and has a major impact on both physical and mental health [1]. Acne pathogenesis is multi-factorial, with both extrinsic and intrinsic contributions. The conventional description of the pathogenesis has focused on local factors in relation to the skin, such as overactive sebum production, follicular hyperkeratinization, overgrowth of *Cutibacterium acnes* (*C. acnes*), and inflammation [2]. Typically, acne presents with inflammatory lesions on the face, shoulders, and chest, along with non-inflammatory lesions such as open or closed comedones [3,4].

Recent evidence shows that systemic and distant factors may have an influence on acne as well. Some examples include noted associations with circulating androgens [5,6,7], shifts in circulating short-chain fatty acids [8], and shifts in the gut microbiome [9]. In particular, previous studies have noted that circulating hormones such as testosterone, progesterone, dihydrotestosterone, and estrogen modulate acne [10,11,12]. Several therapies improve acne by modulating the hormones, with examples such as oral and topical spironolactone [13,14], topical clascoterone [15,16], oral contraceptive pills [17], or oral soy isoflavones [12]. In relation to stress, multiple studies show that elevated stress correlates with worsening of acne [18]. Moreover, excessive cortisol has been associated with the presence of acne either in the absence [19] or presence [20] of a tumor leading to androgen and cortisol excess. However, cortisol correlations to specific lesion counting have not been performed.

The gut microbiome appears to have an influence on acne as well. For example, shifts in the gut microbiome have also been associated with either the presence or absence of acne [21,22]. In further support for the role of the gut microbiome, several prospective interventional studies with oral probiotics have improved acne [8,23,24].

While these previous studies have looked at correlations in relation to either the presence or absence of acne, there have not been any specific correlative studies that have correlated specifically against inflammatory or non-inflammatory lesion counts or against the skin’s biophysical features. Here, we explore the association between circulating hormones and the gut microbiome to acne and correlate it specifically to the presence of either non-inflammatory or inflammatory lesions of acne to better understand the correlations to acne.

## 2. Methods

### 2.1. Subjects

This study was conducted between March 2022 and December 2022 as an 8-week open-label study. Institutional Review Board approval was received on 5 March 2022, by Allendale, and the study was listed on clinicaltrials.gov (NCT05271487). Written informed consent was received from all participants prior to enrollment. Subjects from the greater Sacramento region were recruited. Inclusion criteria included males and females between the ages of 12 to 45 years old, with mild-to-moderate acne classified by an investigator global assessment (IGA) of 2 or 3, along with the presence of at least 10 inflammatory lesions and at least 15 total acne lesions. Subjects who had more than two nodules were excluded. Those with severe acne (IGA ≥ 4), women who were pregnant or breastfeeding, those who were current smokers or had a smoking history of >10 pack years, those unwilling to discontinue facial products except for what is provided in the study, and those who changed their hormonal based contraception within 3 months prior to enrollment were excluded from the study. Those who had isotretinoin use within the 3 months prior to joining the study and those who were unable to discontinue oral antibiotics, probiotics, topical antibiotics, and topical benzoyl peroxide use were also excluded from the study.

### 2.2. Study Visits and Procedures

Written consent and assent were obtained prior to enrollment. Subjects were asked to undergo a 2-week washout from topical antibiotics or benzoyl peroxide use, or a 4-week washout for oral probiotic supplements or oral antibiotic use. The main study reported previously consisted of a total of 4 visits (a screening visit, a baseline visit, a visit after 4 weeks of product use, and after 8 weeks of product use) [25]. This manuscript focuses on correlations from the baseline visit prior to any exposure to investigational product.

Facial and truncal lesion counts for inflammatory and non-inflammatory lesions were performed by a trained doctor or board-certified dermatologist. During these visits, biophysical features such as stratum corneum hydration, trans-epidermal water loss, and sebum excretion rate were obtained using the MoisturemeterSC^®^ (Delfin Technologies, Kuopio, Finland), Vapometer^®^ (Delfin Technologies) and the Sebumeter^®^ (Courage+Khazaka electronic GmbH, Köln, Germany), respectively.

Circulating hormones were assessed with kits supplied by ZRT Laboratories (Beaverton, OR, USA); measurements included a four-point salivary cortisol test, testosterone, progesterone, estradiol, and dehydroepiandrosterone sulfate (DHEA-S). A normalized peak-to-trough difference in the cortisol was utilized to measure cortisol variability, where the absolute peak-to-trough difference in the cortisol levels was normalized against the peak cortisol level.

### 2.3. Gut Microbiome Sequencing and Analysis

Stool samples were collected with sample collection kits, and the stool was stored at −80 °C until they were shipped for analysis on dry ice. Taxonomic and functional analyses of the WGS stool samples were performed by CosmosID.

DNA from fecal samples was isolated using the QIAGEN DNeasy PowerSoil Pro Kit, according to the manufacturer’s protocol. Extracted DNA samples were quantified using Qubit 4 fluorometer and Qubit™ dsDNA HS Assay Kit (Thermofisher Scientific, Waltham, MA, USA). Isolates were stored at −80 °C until they were sequenced.

DNA libraries were prepared using the Nextera XT DNA Library Preparation Kit (Illumina, San Diego, CA, USA) and IDT Unique Dual Indexes with total DNA input of 1 ng. Genomic DNA was fragmented using a proportional amount of Illumina Nextera XT fragmentation enzyme. Unique dual indexes were added to each sample, followed by 12 cycles of PCR to construct libraries. DNA libraries were purified using AMpure magnetic Beads (Beckman Coulter, Brea, CA, USA) and eluted in QIAGEN EB buffer. DNA libraries were quantified using Qubit 4 fluorometer and Qubit™ dsDNA HS Assay Kit (Thermofisher Scientific, Waltham, MA, USA). Libraries were then sequenced on an Illumina HiSeq 4000 platform 2 × 150 bp with a read depth of 3 million reads per sample.

Unassembled sequencing reads were directly analyzed by CosmosID-HUB Micro-biome Platform (CosmosID Inc., Germantown, MD, USA) as described elsewhere for multi-kingdom microbiome analysis, the profiling of antibiotic resistance and virulence genes, and quantification of organisms’ relative abundance [26,27,28,29]. Briefly, the system utilizes curated genome databases and a high-performance data-mining algorithm that rapidly disambiguates hundreds of millions of metagenomic sequence reads into the discrete microorganisms, engendering the particular sequences with the assistance of HUMAnN 3.0 software. Similarly, the community resistome and virulome, the collection of antibiotic resistance and virulence-associated genes in the microbiome, were also identified by querying the unassembled sequence reads against the CosmosID curated antibiotic resistance and virulence-associated gene databases.

### 2.4. Statistical Analysis

The statistical analysis was performed using Pearson Correlations and associated *p*-values. The correlations were performed in Python and used a set of nested loops with the actual Pearson Correlation and *p*-values calculated using SciPy.Stats and the pearsonr(arg1, arg2) function. (https://docs.scipy.org/doc/scipy-1.2.3/reference/generated/scipy.stats.pearsonr.html, last accessed 6 August 2023). Because of the pilot nature of this study, we stratified the *p*-values to seek trends in addition to statistical significance. A *p*-value of less than 0.3 was determined to be a weak correlation, a *p*-value less than 0.2 was considered a moderate correlation, and a *p*-value less than 0.1 was considered to be a strong correlation.

## 3. Results

Twenty-four participants were screened, and twenty participants completed all baseline visits and lab collection procedures. Three participants did not complete the lab collection procedures per protocol, and a total of seventeen were analyzed. The majority of participants were female, with a mean age of 24.4 ± 7.3 years. The mean acne severity at enrollment was graded to be an IGA of 2.7.

### 3.1. Correlations to Facial Acne Lesion Counts

Facial non-inflammatory lesions were weakly correlated to the cheek sebum excretion rate (corr: 0.145, *p*-value: 0.28), inversely weakly correlated to forehead hydration (corr: −0.276, *p*-value: 0.13), and inversely weakly correlated to cheek hydration (corr: −0.170, *p*-value: 0.25).

### 3.2. Correlations to Skin Biophysical Properties

The facial sebum excretion rate was weakly correlated to the circulating levels of testosterone (corr: 0.24, *p*-value: 0.17), inversely correlated to the progesterone/testosterone ratio (corr: −0.35, *p*-value: 0.081), and inversely weakly correlated to the normalized peak-to-trough cortisol difference (corr: −0.18, *p*-value: 0.26).

The facial hydration was correlated with circulating levels of estradiol (corr: 0.41, *p*-value: 0.042) and with the normalized peak-to-trough cortisol difference (corr: 0.40, *p*-value: 0.061).

### 3.3. Correlations to the Gut Microbiome

Multiple gut bacteria either inversely or directly correlated with non-inflammatory lesions (Table 1). Two pathogenic bacteria that were correlated with facial non-inflammatory counts included *Actinomyces naeslundii* (corr: 0.54, *p*-value: 0.013) and *Intestinibacter bartlettii* (corr: 0.46, *p*-value: 0.041), while the bacterium that was most inversely correlated was *Adlercreutzia equolifaciens* (corr: −0.61, *p*-value: 0.0039), which is an equol (isoflavone)-producing bacterium [30].

In regard to the inflammatory lesions of acne, several bacteria were correlated with the presence of acne (Table 2) such as *Clostridium* (corr: 0.74, *p*-value: 0.0001), *Butyrivibrio crossotus* (corr: 0.72, *p*-value: 0.00036), *Pseudoruminococcus massiliensis* (corr: 0.72, *p*-value: 0.00036), *Sutterella faecalis* (corr: 0.72, *p*-value: 0.00036), *Megamonas rupellensis* (corr: 0.71, *p*-value: 0.00040), *Akkermansia muciniphila* (corr: 0.71, *p*-value: 0.00040), and *Methanobrevibacter smithii* (corr: 0.53, *p*-value: 0.016).

When assessing for particular strains, the following strains were negatively correlated with non-inflammatory lesions (Table 3): *Actinomyces naeslundii str Howell 279*, *Bifidobacterium dentium*, *Intestinibacter bartlettii DSM 16795*, and *Eubacterium sp AM28-29.* The following strains were inversely correlated to non-inflammatory lesions (Table 3): *Blautia obeum ATCC 29174*, *Massilioclostridium coli*, *Schaalia odontolytica*, *Adlercreutzia equolifaciens subsp celatus*, and *Butyricicoccus sp GAM44.*

In regard to the inflammatory lesions, the bacterial strains that positively correlated with inflammatory lesions are shown in Table 4, and the notable ones include the following: *Coprococcus sp AF16-22*, *Butyrivibrio crossotus DSM 2876, Clostridium sp AF23-8, Escherichia coli KTE51, Akkermansia muciniphila ATCC BAA-835, Bilophila wadsworthia 316,* and *Methanobrevibacter smithii DSM2375*. The following bacteria were inversely correlated to inflammatory lesions (Table 4): *Coprococcus sp ART55-1* and *Alistipes senegalensis JC50*.

## 4. Discussion

This study explores several correlations to both acne and skin biophysical features in those with acne. Here, we discuss how circulating hormones and stress may correlate to the skin of those with acne. Furthermore, we noted that the presence of certain gut bacteria is correlated with the number of facial and truncal inflammatory lesions. Unlike previous studies that have broadly looked at the presence or absence of acne [9], our study is more granular because we correlate our findings against lesion counting rather than simply the presence or absence of acne.

We show that testosterone levels are correlated to sebum excretion rate. This is in agreement with a previous study that showed that testosterone levels correlated with sebum excretion rate [31,32] and with different severity levels of acne [5]. This is also in agreement with another previous study that correlated elevated testosterone levels to the presence of acne [33]. Interestingly, a different study that correlated against acne severity did not find a correlation between testosterone levels and acne severity [34]. The investigators stratified acne into minor, mild, and moderate acne based on the Global Acne Evaluation scale [35] rather than lesion counting. Therefore, while testosterone appears to correlate with sebum excretion rate, there may be interindividual differences in responsiveness to testosterone.

Our finding that non-inflammatory lesions are weakly inversely correlated with facial hydration offers greater detail in a space that previously had contradictory findings. For example, while decreased epidermal hydration has been identified in association with acne [36], another study identified no epidermal hydration deficits in association with acne [37]. The reason for the discrepancy may be related to the different methodologies used to measure hydration between the two studies. However, our findings suggest that hydration may be more related to comedogenesis, and this suggestion is supported by previous work that identified that ceramides were the deficient lipid in those with acne [36]. Ceramide deficiency is believed to be associated with a decreased water-holding capacity, follicular hyperkeratosis, and comedone formation [36]. Our findings shed more light on this by differentiating the correlations between non-inflammatory and inflammatory lesions and suggesting that the correlation is with comedone formation (non-inflammatory lesions) rather than inflammation (inflammatory lesions).

When assessing the role of stress, we elected to correlate against the normalized peak-to-trough cortisol difference to minimize collection-based errors that may arise with the four-point cortisol measurement when the subject shifts the recommended time points [38,39]. Several findings were notable. The peak-to-trough normalized cortisol ratio was inversely correlated to sebum excretion rate and positively correlated with skin hydration. Because a decreased peak-to-trough ratio is correlated with a more stressed state, our results suggest that a highly stressed state (lower peak-to-trough normalized cortisol ratio) is associated with higher sebum excretion rate and lower skin hydration, which would imply that increased stress can worsen acne.

Previous studies have also noted an association with increased stress and acne [40,41]. While an association between increased stress and increased sebum excretion was not noted in [41], the measurement of self-reported stress was based on the Perceived Stress Scale rather than the objective measure of stress that was used in our study, where we were able to directly correlate an objective measure of stress and sebum excretion rate. Furthermore, our findings that the peak-to-trough normalized cortisol difference is positively correlated to skin hydration are in agreement with previous findings that increased stress is associated with decreased skin barrier function [42,43], and this barrier deterioration leads to dehydration. However, exposure to psychological stress has not been reported to modulate skin hydration in a previous report [44], although this study used a population of healthy women and only performed a single-point analysis of their cortisol levels. Our study differs in that the measurements were among subjects with acne and utilized the normalized peak-to-trough ratio in cortisol, which is less susceptible to single-point fluctuations.

Our results expand on the correlations of the gut microbiome to the presence of acne by specifying the correlations between specific species and strains against specific acne lesion types. One example is the *Butyricoccus* species, which we identified as inversely correlated to non-inflammatory lesions. This bacterium has previously been shown to be deficient in those with acne [9]. Another example, *Intestinibacter bartlettii*, which was correlated to facial non-inflammatory lesions, has also been correlated to increased body mass index and metabolic derangements in obese children [45]. Another microbe uncovered in our analysis was the association of *Actinomyces naeslundii,* which correlated to facial non-inflammatory lesions, and this is typically a result of periodontal disease. Our results are in agreement with other studies that have correlated periodontal disease and with dermatological diseases such as psoriasis and pemphigus [46], but our study establishes the presence of a specific bacteria with the presence of acne-based lesions and warrants further study into this association. On the other hand, the bacteria that had the highest inverse correlation to facial non-inflammatory lesions was *Adlercreutzia equolifaciens*, which is an equol-producing bacteria. Supplementation of isoflavones has previously been shown to both modulate hormones and improve acne [12], and it is worth further follow-up with more studies to better understand the possible influence of *Adlercreutzia equolifaciens* on acne. Another bacterial genus that emerged as correlated with the presence of inflammatory lesions was *Clostridium*, which has been previously identified as a pathogenic genus [47], although it has never been correlated with acne. One previous study found that *Clostridia* depletion may be correlated with acne [21], which appears in opposition to our findings. However, a more specific look at the associations may explain the difference as our data are more granular in that we correlated separately against non-inflammatory and inflammatory lesions, with the finding that *Clostridia* inversely correlated non-significantly to non-inflammatory lesions (corr: −0.23, *p*-value: 0.34) but had a stronger correlation with the presence of inflammatory lesions (corr: 0.74, *p*-value: 0.0001). Furthermore, we noted an association for the presence of inflammatory lesions with *Methanobrevibacter smithii*, which is a known pathogen associated with gut dysbiosis such as Small Intestinal Bacterial Overgrowth (SIBO) and Intestinal Methanogen Overgrowth (IMO) [48]. While SIBO has correlated with the scleroderma and rosacea [22,49], our data suggest that further exploration into the association between SIBO and inflammatory acne may be warranted.

A notable finding is that we identified an inverse correlation between the gut presence of *Akkermansia mucihiphila* and inflammatory lesions. Although *A. muciniphila* presence has typically been associated with decreased inflammation [50,51], several studies have identified possible pro-inflammatory associations with *A. muciniphila* as well [51]. In light of the many anti-inflammatory associations, it remains unclear if *A. muciniphila* is associated with inflammatory lesions as a causative effect or as a reactionary mechanism by the gut microbiota to reduce inflammation.

As the evidence for the role of nutrition in acne continues to grow, there has been increased interest in understanding how the gut microbiome correlates with the presence of acne. Previous gut microbiome analyses have indicated that an increase in the *Proteobacteria/Actinoobacteria* ratio and a decrease in the *Lactobacillus, Bifidobacterium,* and *Butyirococcus* genera are associated with the presence of acne [9]. Another study noted that the gut microbiome was depleted of *Clostridia*, *Clostridiales*, *Lachnospiraceae,* and *Ruminococcaceae* genera in those with acne [21]. In both previous studies, the sequencing was performed through the use of 16S technology, which cannot identify specific strains. Our study utilizes whole genome shotgun sequencing methods, rather than 16S, that allow for more detailed species and strain level data. For example, our study shows different results from the aforementioned study in that the *Clostridum* species positively correlated with inflammatory lesions and, in particular, the strain of Clostridium *Clostridium sp AF 23-8* was correlated to the presence of inflammatory lesions. The reason for the differential results is not clear, but may reflect that there could be a strain-to-strain level variation that may not be captured at the species level. Further studies will help elucidate the contributions of gut bacterial species and strains.

## 5. Conclusion

Our findings suggest that multiple factors may correlate with shifts in the skin’s biophysical properties and with acne lesion counts. In particular, the normalized peak-to-trough cortisol ratio may correlate with facial hydration and inversely correlate with sebum excretion rate. Finally, the gut microbiome correlated with acne with differential correlations between different species with inflammatory and non-inflammatory lesions.

## Figures and Tables

**Table 1:** Gut bacteria species associated with non-inflammatory lesions of acne.

Bacterial Species	Correlation	*p*-Value
**Positive Correlation**		
*Lactobacillus_delbrueckii*	0.583	0.007
*Actinomyces_naeslundii*	0.543	0.013
*Bacteroidales_u_s*	0.535	0.015
*Bifidobacterium_dentium*	0.511	0.021
*Bifidobacterium_u_s*	0.505	0.023
*Bifidobacterium_longum*	0.483	0.031
*Intestinibacter_bartlettii*	0.460	0.041
**Inverse Correlation**		
*Adlercreutzia_equolifaciens*	−0.615	0.004
*Schaalia_odontolytica*	−0.470	0.037
*Massilioclostridium_coli*	−0.458	0.042

**Table 2:** Gut bacteria species associated with inflammatory lesions of acne.

Bacterial Species	Correlation	*p*-Value
**Positive Correlation**		
*Clostridium_u_s*	0.744	0.0002
*Butyrivibrio_crossotus*	0.718	0.0004
*Sutterella_faecalis*	0.718	0.0004
*Pseudoruminococcus_massiliensis*	0.718	0.0004
*Akkermansia_muciniphila*	0.715	0.0004
*Megamonas_rupellensis*	0.714	0.0004
*Prevotella_pectinovora*	0.635	0.0027
*Collinsella_bouchesdurhonensis*	0.616	0.0038
*Hungatella_effluvii*	0.593	0.0058
*Parabacteroides_massiliensis*	0.557	0.0108
*Hungatella_hathewayi*	0.555	0.0110
*Parolsenella_catena*	0.541	0.0137
*Prevotella_stercorea*	0.541	0.0137
*Methanobrevibacter_smithii*	0.532	0.0158
*Roseburia_hominis*	0.505	0.0230
*Phascolarctobacterium_faecium*	0.494	0.0270
**Inverse Correlation**		
*Alistipes_senegalensis*	−0.475	0.034

**Table 3:** Gut bacteria strains associated with non-inflammatory lesions of acne.

Bacterial Strain	Correlation	*p*-Value
**Positive Correlation**		
*Actinomyces_naeslundii_str_Howell_279*	0.543	0.013
*Bacteroidales_u_t*	0.535	0.015
*Bifidobacterium_dentium*	0.511	0.021
*Firmicutes_u_t*	0.461	0.041
*Intestinibacter_bartlettii_DSM_16795*	0.460	0.041
*Eubacterium_sp_AM28-29*	0.460	0.041
**Inverse Correlation**		
*Blautia_obeum_ATCC_29174*	−0.455	0.044
*Massilioclostridium_coli*	−0.458	0.042
*Schaalia_odontolytica*	−0.470	0.037
*Adlercreutzia_equolifaciens_subsp_celatus*	−0.482	0.031
*Butyricicoccus_sp_GAM44*	−0.514	0.020

**Table 4:** Gut bacteria strains associated with inflammatory lesions of acne.

Bacterial Strain	Correlation	*p*-Value
**Positive Correlation**		
*Coprococcus_sp_AF16-22*	0.773	6.53381 × 10^−5^
*Lachnospiraceae_bacterium_OM04-12BH*	0.735	0.000225629
*Butyrivibrio_crossotus_DSM_2876*	0.718	0.000362097
*Clostridium_sp_AF23-8*	0.718	0.000362097
*Escherichia_coli_KTE51*	0.718	0.000362097
*Faecalibacterium_sp_AF28-13AC*	0.718	0.000362097
*Sutterella_faecalis*	0.718	0.000362097
*Bacteria_u_t*	0.718	0.000362097
*Bacteroides_dorei_CL03T12C01*	0.718	0.000362097
*Pseudoruminococcus_massiliensis*	0.718	0.000362097
*Megamonas_rupellensis_DSM_19944*	0.714	0.000404027
*Bacteroides_pectinophilus_ATCC_43243*	0.714	0.00041069
*Akkermansia_muciniphila_ATCC_BAA-835*	0.702	0.00056436
*Bilophila_wadsworthia_3_1_6*	0.653	0.001781736
*Eubacterium_siraeum_70_3*	0.646	0.002113114
*Prevotella_pectinovora*	0.634	0.002658279
*Methanobrevibacter_smithii_DSM_2375*	0.625	0.003234041
*Collinsella_bouchesdurhonensis*	0.616	0.003806916
*Hungatella_hathewayi_VE202-04*	0.611	0.004206782
*Hungatella_effluvii*	0.593	0.005827701
*Collinsella_sp_AM38-1BH*	0.593	0.005888244
*Bifidobacterium_u_t*	0.584	0.006908296
*Parabacteroides_massiliensis*	0.557	0.010799462
*Parolsenella_catena*	0.541	0.013713086
*Prevotella_stercorea_DSM_18206*	0.541	0.013744505
*Bacteroides_vulgatus_str_3975_RP4*	0.526	0.017310217
*Roseburia_hominis_A2-183*	0.505	0.023038589
*Phascolarctobacterium_faecium*	0.494	0.026951749
*Megamonas_sp_Calf98-2*	0.494	0.026973871
*Ruminococcus_sp_AM26-12LB*	0.459	0.041819714
**Inverse Correlation**		
*Bilophila_u_t*	−0.455	0.043759349
*Coprococcus_sp_ART55_1*	−0.456	0.043278258
*Alistipes_senegalensis_JC50*	−0.475	0.034207072

## Data Availability

No publicly archived datasets.
